# Some Remarks on the Supposed Influence of the Moon on Fevers

**Published:** 1787

**Authors:** James Lind

**Affiliations:** Physician at Windsor, and Fellow of the Royal College of Physicians at Edinburgh


### VII. *Some Remarks on the ſuppoſed Influence of the Moon on Fevers. Communicated in a Letter to Dr.* Simmons, *by* James Lind, *M. D., F. R. S. Phyſician at Windſor, and Fellow of the Royal College of Phyſicians at Edinburgh.*

I SEE by the letter of Dr. Jackſon, Phyſician at Stockton, publiſhed in the firſt part of the London Medical Journal for the preſent year, that there are others, beſides the inhabitants of the lower part of Bengal, Dr. Balfour, &c. and myſelf, who have attributed the frequent attacks and returns of remittent and intermittent fevers, which happen in tropical countries about the times of the new and full moon, to the immediate influence of the moon.

I confeſs I was once of this opinion, as you may ſee by my Inaugural Diſſertation on the Marſh Fever, which raged at Bengal in 1762*; but of this immediate influence I have, upon more mature conſideration, long ſince doubted †, and think that it ought rather to be imputed

* Re-publiſhed in the third volume of the Edinburgh Theſaurus Medicus.

† *Vide* page 45 of the Engliſh tranſlation of the above Diſſertation, printed at Edinburgh.

puted to the noxious vapours arifing from the fwamps, produced by the high tides which happen at the time of the full and change of the moon, and, overflowing a great part of the country, leave it in a marfhy ftate at low water, thereby occafioning the frequent attacks and relapfes that occur at thofe periods. This I am induced to believe to be the fole caufe: firft, becaufe this lunar influence entirely ceafes when the patient is removed but a few miles from the fwamps that are left uncovered by the tide at low water; fecondly, becaufe intermittent fevers are not obferved to follow lunar periods at many places within the tropics, even at Canton *, (where there is a large river and great tides) by reafon of the induftrious Chinefe keeping the river within its bounds. Intermittents there only follow the ftate of the weather, as it renders the country and rice grounds more or lefs marfhy, or as the winds blow over dry country, or rice grounds that are covered with mud and flime; therefore, what is called a lunar influence will, I imagine, be no

| | | ° | ′ | ″ | |
|---|---|---|---|---|---|
| * Latitude of Canton in China, | - | 23 | 6 | 38 | N. |
| Latitude of Calcutta in Bengal, | - | 22 | 34 | 45 | |

where found, but where remitting and intermitting fevers are occafioned from muddy fhores left by the ebbing of the tide.

*Windfor,*

April 23d, 1787.

